# Botulinum neurotoxin C mutants reveal different effects of syntaxin or SNAP-25 proteolysis on neuromuscular transmission

**DOI:** 10.1371/journal.ppat.1006567

**Published:** 2017-08-11

**Authors:** Giulia Zanetti, Stefan Sikorra, Andreas Rummel, Nadja Krez, Elisa Duregotti, Samuele Negro, Tina Henke, Ornella Rossetto, Thomas Binz, Marco Pirazzini

**Affiliations:** 1 Department of Biomedical Sciences, University of Padova, Padova, Italy; 2 Institut für Zellbiochemie, Medizinische Hochschule Hannover, Hannover, Germany; 3 Institut für Toxikologie, Medizinische Hochschule Hannover, Hannover, Germany; University of Pittsburgh School of Medicine, UNITED STATES

## Abstract

Botulinum neurotoxin serotype C (BoNT/C) is a neuroparalytic toxin associated with outbreaks of animal botulism, particularly in birds, and is the only BoNT known to cleave two different SNARE proteins, SNAP-25 and syntaxin. BoNT/C was shown to be a good substitute for BoNT/A1 in human dystonia therapy because of its long lasting effects and absence of neuromuscular damage. Two triple mutants of BoNT/C, namely BoNT/C *S51T/R52N/N53P* (BoNT/C α-51) and BoNT/C *L200W/M221W/I226W* (BoNT/C α-3W), were recently reported to selectively cleave syntaxin and have been used here to evaluate the individual contribution of SNAP-25 and syntaxin cleavage to the effect of BoNT/C *in vivo*. Although BoNT/C α-51 and BoNT/C α-3W toxins cleave syntaxin with similar efficiency, we unexpectedly found also cleavage of SNAP-25, although to a lesser extent than wild type BoNT/C. Interestingly, the BoNT/C mutants exhibit reduced lethality compared to wild type toxin, a result that correlated with their residual activity against SNAP-25. In spite of this, a local injection of BoNT/C α-51 persistently impairs neuromuscular junction activity. This is due to an initial phase in which SNAP-25 cleavage causes a complete blockade of neurotransmission, and to a second phase of incomplete impairment ascribable to syntaxin cleavage. Together, these results indicate that neuroparalysis of BoNT/C at the neuromuscular junction is due to SNAP-25 cleavage, while the proteolysis of syntaxin provides a substantial, but incomplete, neuromuscular impairment. In light of this evidence, we discuss a possible clinical use of BoNT/C α-51 as a botulinum neurotoxin endowed with a wide safety margin and a long lasting effect.

## Author summary

The seven established Botulinum Neurotoxins serotypes (BoNT/A to G) and the many BoNT subtypes, the causative agents of botulism, are the most poisonous substances known (lethal doses in the low ng/kg range). Due to their toxicological properties, BoNTs are Janus-faced toxins: potent pathogenic factors and potential bioterrorism agents as well as safe and efficacious therapeutics. BoNTs exert their neuroparalytic action by cleaving SNARE proteins, either SNAP-25 or synaptobrevin/VAMP, which mediate neurotransmitter release at the neuromuscular junction; BoNT/C is the only serotype shown to cleave SNAP-25 and syntaxin-1 *in vitro*. Our study shows for the first time that this parallel cleavage also occurs *in vivo*. By using mutated toxins reported to be syntaxin-selective, we found that SNAP-25 proteolysis at the neuromuscular junction is the key determinant of BoNT/C lethality as it completely blocks nerve-muscle transmission. Conversely, syntaxin-1 cleavage only attenuates nerve terminal activity without inactivating the synapse, leading to only a partial decrease of neuromuscular functionality. As a result, the BoNT/C mutants have dramatically reduced lethality, but still modulate neuromuscular junction activity upon intramuscular injection. This aspect is particularly relevant considering the possible use of syntaxin-specific BoNT/C derivatives to improve the present clinical utilization of BoNTs.

## Introduction

Few species of the bacterial genus *Clostridium* produce botulinum neurotoxins (BoNTs), which cause the flaccid paralysis of botulism [1]. BoNTs are divided into at least seven different serotypes (BoNT/A to G) that comprise an increasing number of subtypes [1–3]. BoNTs are the most poisonous toxins known to date and display lethal doses in the low ng/kg range [4, 5]. This remarkable potency is due to their selective action within the peripheral nervous system, most notably at the neuromuscular junction (NMJ), where BoNTs inactivate the machinery responsible for neurotransmitter release, causing muscle paralysis and blockade of autonomic innervations [6]. Therefore, BoNTs are used to treat human diseases characterized by hyperactivity of peripheral nerve terminals of both the motor and autonomic nervous system [7]. This clinical use is almost exclusively restricted to BoNT/A1 as it produces the longest effect, and in very few circumstances to BoNT/B1, mainly to overcome BoNT/A1 resistance [5, 8].

The BoNT structure is composed of three domains that perform different functions [1]: a) the C-terminal part harbors two binding sites for two different receptors that mediate toxin anchoring and internalization within nerve terminals [9, 10]; b) an intermediate domain responsible for the translocation of the catalytic domain into the cytosol of nerve terminals [11, 12]; and c) the N-terminal catalytic domain, termed light chain (LC), which is a metalloprotease cleaving one of the three SNARE (Soluble NSF Attachment Protein Receptors) proteins, namely VAMP-1/2 (vesicle-associated membrane protein 1/2, also known as synaptobrevin-1/2), SNAP-25 (synaptosomal-associated protein of 25 kDa) and syntaxin-1A/1B (Stx) [13, 14]. These three proteins assemble into a complex, i.e. the SNARE complex, which mediates the fusion of synaptic vesicles with the presynaptic membrane [15], and their proteolysis is directly responsible for the pathogenicity of BoNTs [1, 6]. BoNT/B, /D, /F and /G cleave the vesicular SNARE protein VAMP-1/2 [16–19], whereas BoNT/A, and /E cleave the plasma membrane protein SNAP-25 [20, 21]. BoNT/C is the only toxin known to cleave two SNARE substrates, SNAP-25 and syntaxin-1A/1B, *in vitro* [22–24]. Each toxin cleaves its SNARE at a unique site thereby removing different portions of the respective substrates [13, 14]. Interestingly, while BoNTs cleaving VAMP cause a paralysis of intermediate duration, the three serotypes that cleave SNAP-25 provide the shortest and the longest persistence of action [25–28]: BoNT/E removes 26 amino acids from SNAP-25 C-terminus and produces a muscle paralysis of a few days. BoNT/A and BoNT/C remove only nine and eight amino acids, respectively, and cause a paralysis that lasts for months in humans [29–31]. However, it is currently unknown whether BoNT/C cleaves syntaxins at the NMJ and to what extent this cleavage contributes to its long lasting paralysis.

Recently, two BoNT/C LC mutants were reported to display selective protease activity against syntaxins [32]. These mutants offer the unique opportunity of dissecting the contribution of syntaxin and SNAP-25 cleavage to BoNT/C-induced paralysis and duration of action. Therefore, we synthesized the respective full-length BoNT/C mutants and tested their potency *in vitro* and *in vivo*. Surprisingly, we found that the two mutant toxins are much less toxic than wild type BoNT/C and their respective toxicity correlates with an unexpected residual activity against SNAP-25. Our findings suggest that BoNT/C lethality is mainly due to SNAP-25 cleavage, while the proteolysis of syntaxin accounts for a prolonged and substantial, albeit incomplete, impairment of neuromuscular transmission.

## Results

### BoNT/C mutants retain a residual activity on SNAP-25

Based on the work of Wang *et al*. (2011) [32], we produced the full-length triple mutants BoNT/C *S51T/R52N/N53P* (hereafter referred to as BoNT/C α-51) and BoNT/C *L200W/M221W/I226W* (BoNT/C α-3W) in *Escherichia coli*, along with wild type BoNT/C (BoNT/C-wt). Amino acid substitutions are mapped in the crystal structure of BoNT/C-wt LC (PDB 2QN0) and shown in S1 Fig. Mutations are concentrated either in the S1’ pocket of the LC (*L200W/M221W/I226W*, red spot), thus likely impinging on LC-substrate recognition around the active site (blue spot), or on a region outside the active site (*S51T/R52N/N53P*, green spot), which is possibly involved in LC-SNAREs interaction [32]. Recombinant toxins were expressed in *E*. *coli*, activated into di-chain toxins by host proteases, and one-step affinity-purified using StrepTactin-sepharose matrix. The level of purification was suitable for biochemical and toxicological characterization (S2 Fig).

**Fig 1:**
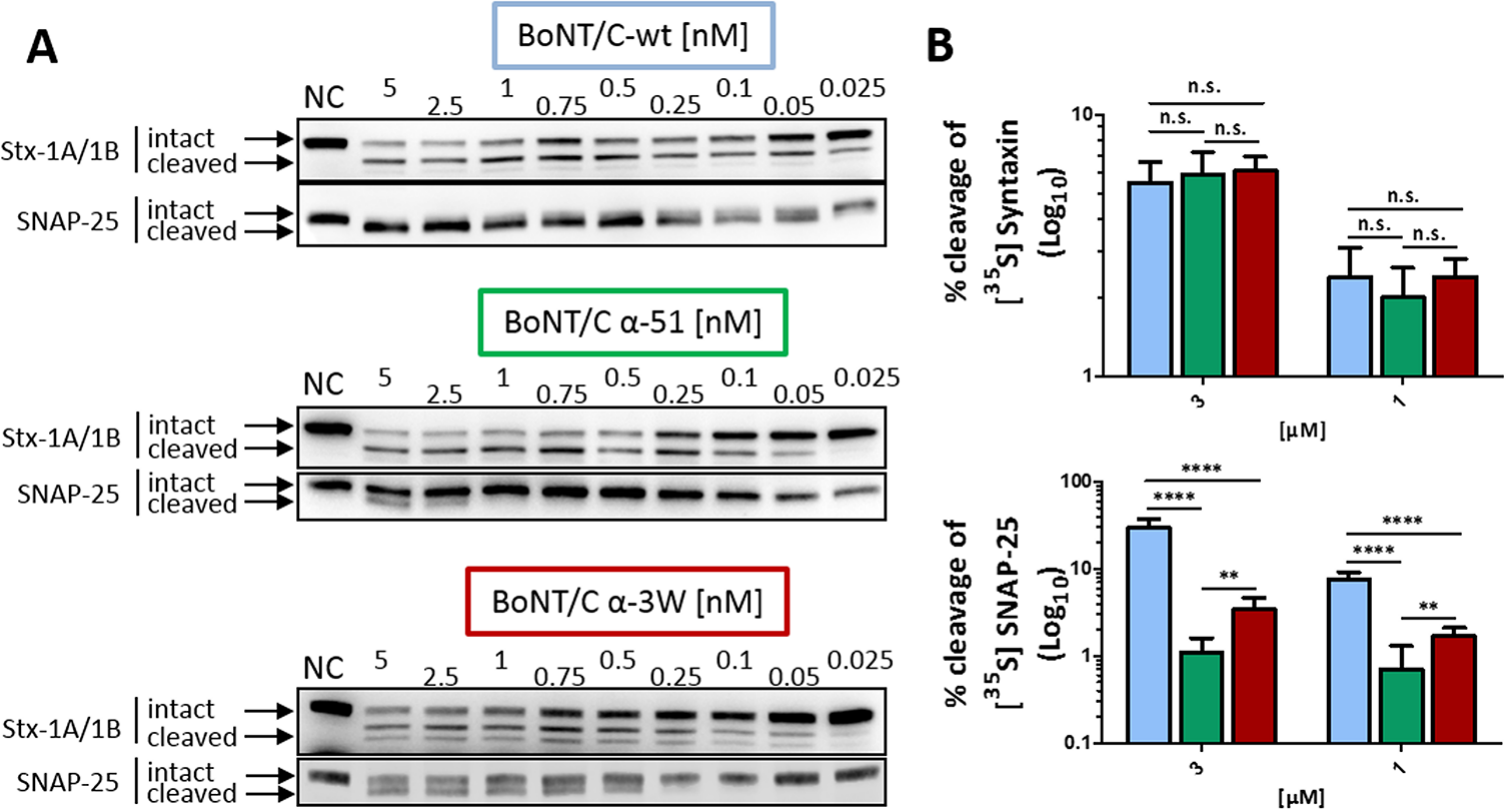
BoNT/C triple mutants retain a residual activity against SNAP-25. **(A)** Proteolytic activity of BoNT/C variants in cultured cerebellar granule neurons (CGNs). Full-length BoNT/C-wt or BoNT/C α-51 or BoNT/C α-3W were added to cultured CGNs at indicated concentrations for 12 hours. The cleavage of syntaxin-1A/1B and SNAP-25 was assayed in western blot by using two antibodies recognizing both the intact and the cleaved forms of the proteins. **(B)** Recombinant syntaxin 1A fusion protein (1 μM, upper panel) or SNAP-25 (10 μM, lower panel) spiked with the corresponding radiolabeled protein generated by *in vitro* transcription/translation in the presence of [^35^S]-Met were incubated with various concentrations of LC/C-wt (cyan) or its mutants (α-51, green; α-3W, red) in toxin assay buffer. After 1 h of incubation at 37°C, samples were analyzed by SDS-PAGE. Percentage of cleavage was quantified by means of the radiolabeled substrate by phosphorimaging. Data are mean values of three to six independent experiments. Statistical significance was determined by a Student's t-test comparing the mean values between groups (* p<0.05, ** p<0.01, *** p<0.001, **** p<0.0001, n.s. not significant).

We first tested the overall functionality of recombinant wild type and mutant BoNT/C in cerebellar granular neurons (CGNs), which are highly sensitive to BoNTs and provide a rapid and reliable method to assay the cleavage of SNARE proteins by western blot [28, 33]. We used two specific antibodies that recognize both the intact and the truncated form of SNAP-25 and syntaxin-1A/1B. After 12 hours of incubation, BoNT/C-wt cleaved both syntaxin-1A/1B (EC_50_ ~ 0.25 nM) and SNAP-25 (EC_50_ ~ 0.05 nM), the latter more efficiently (Fig 1A). Importantly, the extent of cleavage was similar to that of a “natural BoNT/C” purified from *Clostridium botulinum*, implying that production in *E*. *coli* provides BoNT/C-wt with identical biological properties, as previously reported for other recombinant toxins [28, 34–36]. The two mutant BoNT/C toxins cleaved syntaxin-1A/1B with similar efficiency (EC_50_ ~ 0.5 nM), which was only slightly lower compared to BoNT/C-wt, indicating that the mutations do not alter the mutant’s capacity to enter neurons. Contrary to what was reported in the original paper by Wang *et al*. [32], we also detected cleavage of SNAP-25 (Fig 1A, middle and bottom panels), with BoNT/C α-3W being more active (EC_50_ ~ 2.5 nM) than BoNT/C α-51 (EC_50_ > 5 nM) indicating they are not specific for syntaxins. Notably, mutant BoNT/C toxins retained a 50-fold and >100-fold lower activity for SNAP-25 cleavage compared to BoNT/C-wt. This unexpected result may be due to the different methods used, i.e. Wang *et al*. virally transduced the gene encoding for mutant LCs while we exogenously added full-length toxins to the neuron culture medium allowing uptake of physiological amounts. We also noticed that a certain amount of syntaxin-1A/1B appears to be inaccessible to the three BoNT/Cs, even when toxins were used at high concentrations. This became particularly evident when the incubation time was extended to 24 hours (S3 Fig). Moreover, unlike BoNT/C-wt, the amount of SNAP-25 cleaved by BoNT/C α-51 and BoNT/C α-3W did not increase significantly from 12 to 24 hours (Fig 1A and S3 Fig middle and bottom panels).

To further characterize the enzymatic properties of the mutated toxins, we assayed the proteolytic activity of their LCs *in vitro* against recombinant SNAP-25 and syntaxin1A. When applied at equal concentrations, LC/C-wt, LC/C α-51 and LC/C α-3W cleaved syntaxin to a similar extent (Fig 1B, upper panel), suggesting an overall similar enzymatic efficiency. On the other hand, the activity of LC/C α-51 and LC/C α-3W against SNAP-25 (bottom panel) was much lower (30-fold and 10-fold, respectively) than that of LC/C wt.

Collectively, these experiments demonstrate that mutant BoNT/C toxins are not specific for syntaxin-1A/1B, but maintain a residual activity against SNAP-25 which is higher for BoNT/C α-3W compared to BoNT/C α-51.

### BoNT/C mutants have different neurodegenerative effects on neurons

**Fig 2:**
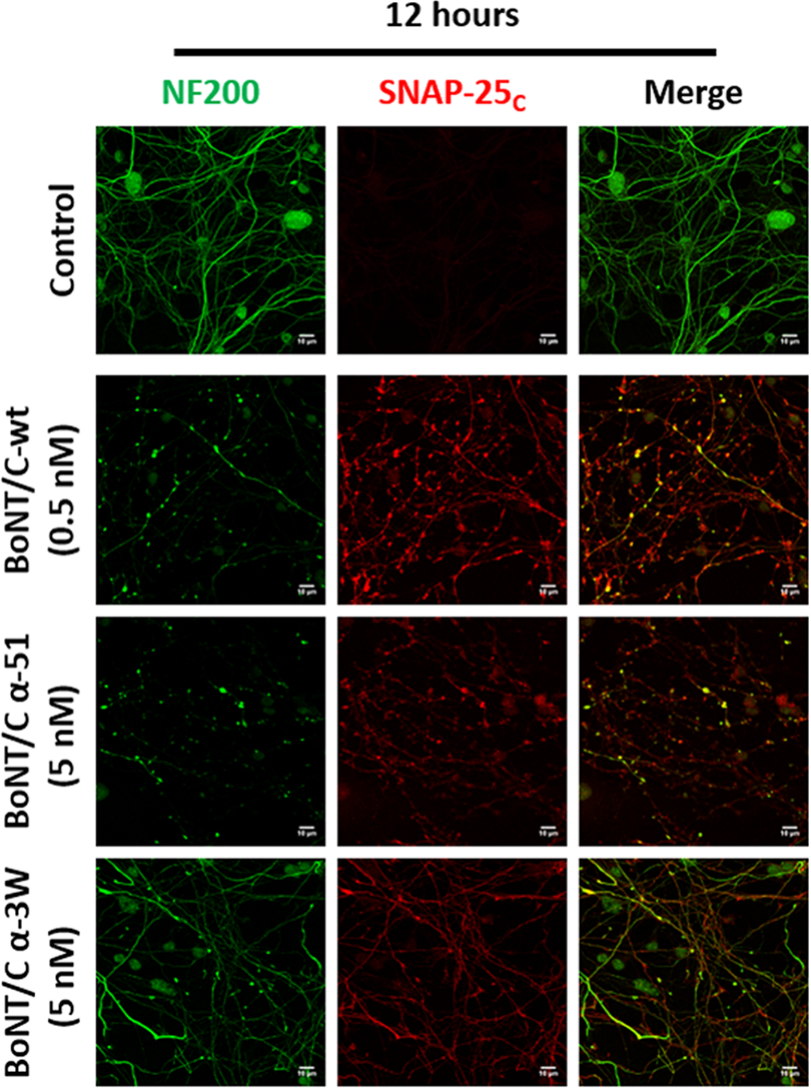
BoNT/C mutants display a different neurodegenerative effect on cultured neurons. CGNs were treated with the indicated concentrations of BoNT/C-wt or mutants for 12 hours. Neurons were then fixed and stained with an antibody against cleaved SNAP-25 (SNAP-25_c_, in red) and neurofilament-200 (NF200, in green). Neurodegeneration was evaluated following the appearance of varicosities along neurites and the loss of NF200 staining. Images are representative of at least three independent experiments. Scale bar, 10 μm.

To provide additional evidence on the proteolytic activity of BoNT/C mutants on SNAP-25, we used an antibody that recognizes SNAP-25 segment 185–197, which corresponds to the newly formed C-terminus generated upon cleavage by BoNT/A. This antibody recognizes BoNT/A-cleaved, and not intact, SNAP-25 both *in vitro* and *in vivo* (S4 Fig) [27, 37, 38]. Since BoNT/C cleaves SNAP-25 one amino acid downstream of the BoNT/A cleavage site [13, 31], we asked whether this antiserum would also recognize BoNT/C-cleaved SNAP-25. The antibody recognized SNAP-25 cleaved by BoNT/C-wt as it caused an accumulation of staining like that generated by BoNT/A1 (S4 Fig). Similar results were obtained upon treatment of CGNs with BoNT/C mutants (12 hours, 5 nM) (Fig 2). In agreement with the western blot analysis, neurons treated with BoNT/C α-3W displayed a more prominent staining compared to those neurons treated with BoNT/C α-51, yet less intense than that arising from BoNT/C-wt treatment (12 hours, 0.5 nM). Moreover, prolonging the incubation time did not significantly increase the amount of cleaved SNAP-25 (S5 Fig).

BoNT/C is long known to cause neurodegeneration in neuronal cultures [39–41], and this effect can be monitored by staining neurofilament-200 (NF200), a major component of axon cytoskeleton. After 12 hours of incubation, BoNT/C-wt caused a substantial loss of NF200 and the formation of varicosities, also filled with cleaved SNAP-25, all along the neurites (Fig 2). BoNT/C α-51 induced a very similar phenotype and caused an evident degeneration of neurons. Intriguingly, neurons treated with BoNT/C α-3W neither displayed significant signs of degeneration nor loss of NF200. This was even more clear after 24 hours of intoxication when the detrimental effects of BoNT/C-wt and BoNT/C α-51 completely degenerated neurons (S5 Fig).

Together with the western blotting analyses, these results suggest that the three BoNT/C variants have different neurodegenerative activity on CGNs and that this effect may not depend on the proteolysis of SNAP-25 and syntaxin-1A/1B.

### BoNT/C mutants are less toxic than wild type BoNT/C

**Fig 3:**
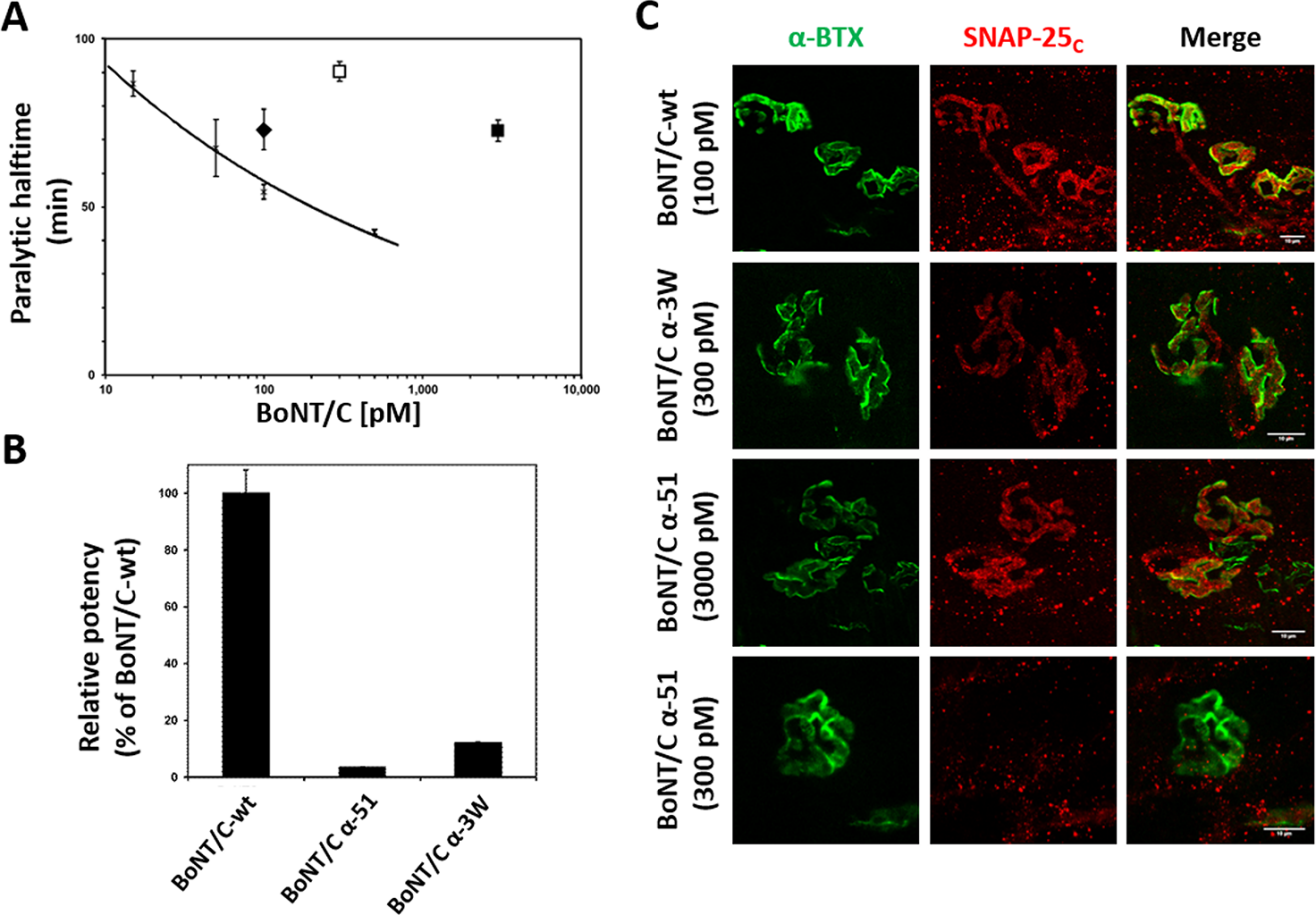
BoNT/C mutants display noticeable lower potency than wild type BoNT/C. **(A)** Activity of BoNT/C variants at the MPN hemidiaphragm assay. The black trace represents a dose-response calibration curve reporting the T_50_ value obtained at indicated bath concentration of a reference wild type BoNT/C [45]. Recombinant BoNT/C-wt (black diamond), tested at 100 pM displays a T_50_ comparable to the previous BoNT/C-wt used at the same concentration. BoNT/C α-3W (white square) and BoNT/C α-51 (black square) need much higher concentrations to achieve a T_50_ within the calibration curve. Error bars represent SD of n = 3–4 technical replicates. **(B)** Calculation of potency of BoNT/C mutants employing a power function fitted to the dose-response calibration curve in A. **(C)** Immunofluorescent analysis of hemidiaphragms derived from MPN assays. Hemidiaphragms treated with the indicated toxin and concentration were fixed immediately upon completion of paralysis and stained for cleaved SNAP-25 (SNAP-25_c_, red). NMJs were spotted with α-Bungarotoxin (α-BTX, in green). Images shown are representative of at least three independent experiments. Scale bar, 10 μm.

Since the three toxins tested here have a similar activity against syntaxin-1A/1B and vary only for the extent of SNAP-25 cleavage, we evaluated the contribution of SNAP-25 cleavage to BoNT/C neuroparalysis. We first assessed the potency of the three toxins using the mouse phrenic nerve hemidiaphragm (MPN) assay. This *ex vivo* assay mimics the respiratory failure of botulism by intoxicating an explanted hemidiaphragm and allows for the recording of muscle contraction capacity elicited by phrenic nerve stimulation [35, 42–45]. The addition of BoNTs to the organ bath impairs nerve-muscle transmission and causes progressive muscle neuroparalysis. The time needed to halve the initial twitch amplitude at a given concentration (T_50_) is proportional to toxin potency [43] and can be used to provide very accurate comparisons of the activity of different BoNT preparations, including mutant toxins [35, 46]. The black trace of Fig 3A shows the T_50_ values obtained for different bath concentrations of a standard BoNT/C, and provides a dose-response calibration curve (y = 148.95x^-0.2089^; R^2^ = 0.9806) [45] which was used to compare the BoNT/C-wt and the two mutant toxins. BoNT/C-wt (100 pM; black diamond) displayed a potency similar to standard BoNT/C (Fig 3A), whereas to achieve a T_50_ value within the calibration curve, BoNT/C α-3W (empty square) and BoNT/C α-51 (filled square) were used at much larger concentrations (300 pM and 3000 pM, respectively). Their potency was calculated as 12.1% and 3.4% of the BoNT/C-wt, respectively (Fig 3B). Hemidiaphragms were then stained for cleaved SNAP-25 (Fig 3C). Cleaved SNAP-25 was detected in all muscles analyzed, suggesting that mutant toxins cleave SNAP-25 also at the NMJ. At the same time, we detected minute levels in hemidiaphragms treated with a concentration of BoNT/C α-51 (300 pM) which caused a very slow decline in muscle strength compared to the higher concentration. These experiments suggest a strict correlation between the potency of BoNT/C variants and their capacity to cleave SNAP-25.

**Fig 4:**
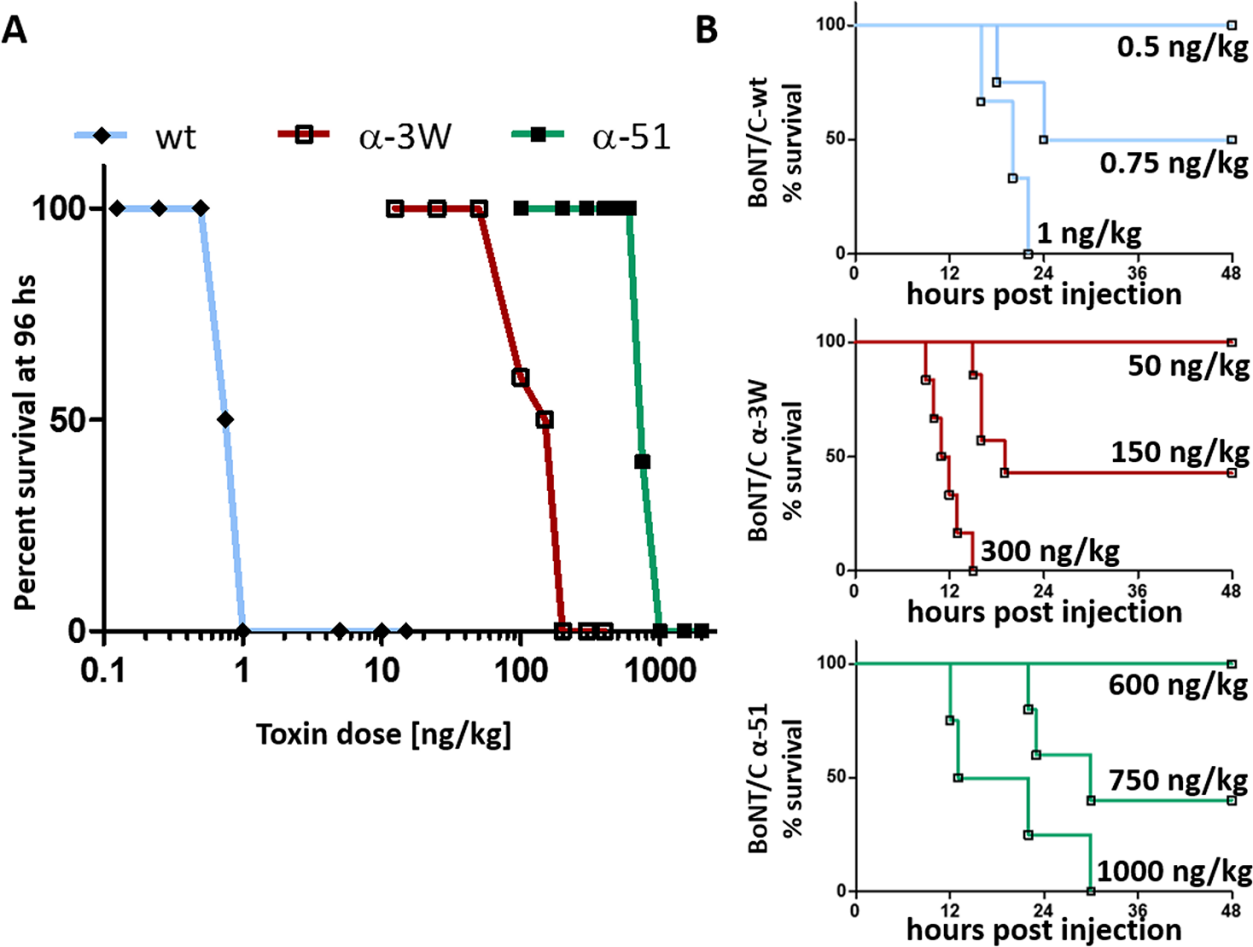
BoNT/C mutants are poorly lethal *in vivo*. **(A)** Mouse bioassay for BoNT/C variants. CD1 female mice weighting 20–24 grams were injected intraperitoneally with the indicated doses of BoNT/C-wt (cyan) or BoNT/C α-51 (green) or BoNT/C α-3W (red). Survival after 96 hours is reported as the percentage of mice survived with respect to the total group treated with the same amount of toxin. **(B)** Animals of the mouse bioassay were monitored every 4 hours and their survival reported as a Kaplan-Meier plot. Top panel shows BoNT/C-wt, middle panel is for BoNT/C α-3W and bottom panel is for BoNT/C α-51.

We next assessed the potency of wild type and mutant BoNT/C toxins *in vivo* using the mouse bioassay [47], in which mice are injected intraperitoneally with different amounts of BoNTs and the dose causing death in the 50% of animals (i.e. 1 LD_50_) is used as a parameter to estimate toxin lethality. BoNT/C-wt resulted in a LD_50_ = 0.75 ng/kg, whereas BoNT/C α-3W and BoNT/C α-51 displayed LD_50_ = 150 ng/kg and LD_50_ = 750 ng/kg, respectively (Fig 4A and Table 1). Similarly to BoNT/C-wt, both mutants induced the classical symptoms of botulism, i.e. the progressive collapse of flank musculature, ruffled fur, eye dryness, and labored breath until respiratory failure. Relative to BoNT/C-wt lethality (Fig 4B, top panel), botulism onset due to BoNT/C α-3W was very rapid as mice died very quickly (Fig 4B, middle panel). Conversely, BoNT/C α-51 induced botulism with a slower progression, with lethality occurring significantly later (Fig 4B, bottom panel). Taken together, these observations suggest that the lethality/potency of the three toxins are linked to their capacity to cleave SNAP-25.

**Table 1:** Summary of the lethality of the different BoNT/C variants used in this study.

	LD_50_	Lethal amount*	Relative lethality
BoNT/C-wt	0.75 ng/kg	0.015 ng	1
BoNT/C α-3W	150 ng/kg	3 ng	1/200
BoNT/C α-51	750 ng/kg	15 ng	1/1000

A comparison of the lethal doses and the lethal amounts of BoNT/C-wt, BoNT/C α-3W and BoNT/C α-51, as estimated from the mouse bioassay. The relative lethality of BoNT/C α-3W and BoNT/C α-51 with respect to BoNT/C-wt is also shown.

(*) The lethal amount is calculated considering a mouse of 20 grams.

### Duration of neuroparalysis does not correlate with potency of mutant BoNT/C toxins

**Fig 5:**
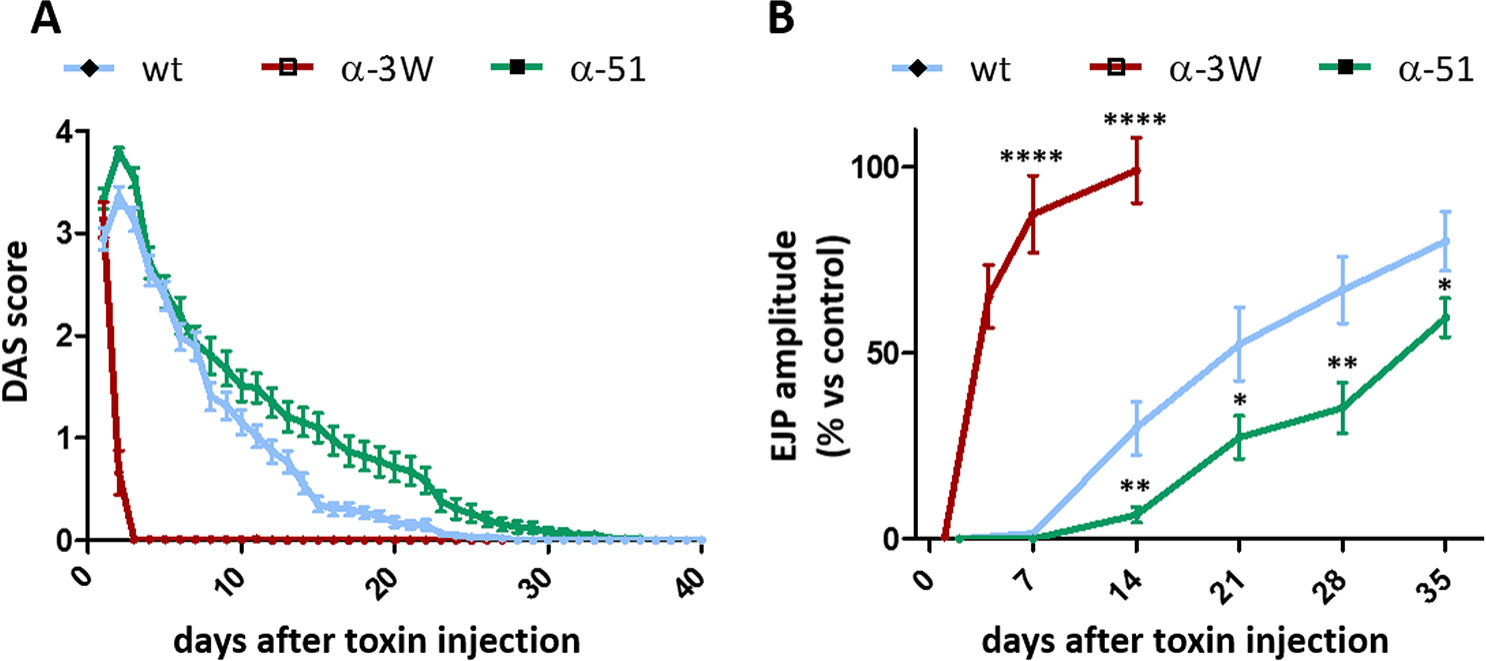
Time course of neuroparalysis recovery upon a local injection of BoNT/C variants in the mouse hind limb. **(A)** Digit Abduction Score (DAS) assay. 1 LD_50_ of BoNT/C-wt (cyan), or BoNT/C α-51 (green) or BoNT/C α-3W (red) were injected intramuscularly in the mice hind limb and neuroparalysis was evaluated according to [49]. The rescue from paralysis was monitored daily until complete recovery was attained. Traces are representative of three independent experiments with at least 5 mice per condition. Error bars represent SEM **(B)** Analysis of evoked post synaptic junction potentials (EJP) on injected soleus muscles. Mice were treated as in A and at indicated time points soleus muscles were collected and processed for recordings of EJPs, as previously reported [27]. Data are presented as a percentage of EJPs of control muscles. Each point represents an average EJP amplitude obtained from at least 45 muscle fibers from three different mice per condition. Statistical significance at each time point was determined by a Student's t-test comparing the mean values between either BoNT/C α-51 (green) or BoNT/C α-3W (red) compared to BoNT/C-wt (cyan) (* p<0.05, ** p<0.01, *** p<0.001, **** p<0.0001, n.s. not significant). Error bars represent SEM.

A fundamental feature of BoNT/C is the long lasting paralysis induced following local injection in sub-lethal amounts. Among the toxins tested to date, BoNT/A1 and BoNT/C have the longest persistence both in human and in mice [26, 48]. Accordingly, we tested the duration of paralysis by mutant toxins upon injection in the hind limb with the Digit Abduction Score (DAS) [49]. In this assay, animals are scored from 0 to 4, where 0 corresponds to normal mobility and 4 to complete block of digit abduction. One LD_50_ of BoNT/C-wt induced a severe paralysis within the first few days after administration, which was then progressively recovered (Fig 5A). Surprisingly, one LD_50_ of BoNT/C α-3W induced a very quick onset followed by a complete recovery within 48 hours. Even more surprisingly, one LD_50_ of BoNT/C α-51 had a very long persistence, also exceeding that of BoNT/C-wt. Interestingly, we detected an acute phase (days 1–5) of severe paralysis followed by a longer period of time characterized by a progressive, yet slow, recovery of function.

To obtain a more quantitative time course of the paralyses, we measured the “evoked junction potential” (EJP). This electrophysiological analysis allows an accurate estimation of neurotransmitter release at the NMJ and can be used to monitor neurotransmission recovery in a quantitative way [27]. Muscles treated with BoNT/C α-3W were fully paralyzed 24 hours after injection, yet recovery was fast and nerve-muscle transmission reached control levels within two weeks (Fig 5B). Unlike BoNT/C α-3W, BoNT/C-wt and BoNT/C α-51 completely abrogated neurotransmission for at least one week and recovery was slow, being incomplete even after 5 weeks.

Altogether, these experiments indicate that the duration of the neuroparalytic effects of BoNT/C mutants does not correlate with their respective potency.

### Cleavage of SNAP-25 or syntaxin-1B have different functional consequences on neuromuscular transmission

**Fig 6:**
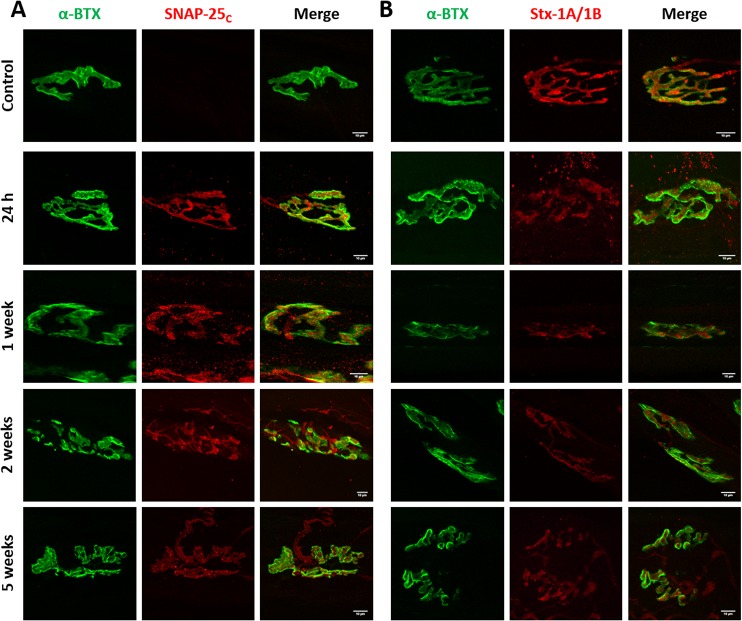
Imaging of cleaved SNAP-25 and syntaxin-1 in muscles treated with BoNT/C-wt. Soleus muscles of mice treated with BoNT/C-wt and used for the analysis of EJPs of Fig 5B were fixed immediately after the electrophysiological recordings and stained for **(A)** cleaved SNAP-25 (SNAP-25_c_) or **(B)** Syntaxin-1A/1B (Stx-1A/1B), both shown in red. NMJs were spotted with α-Bungarotoxin (α-BTX, in green). The first row of panels represents the staining of a control muscle. Scale bar, 10 μm.

**Fig 7:**
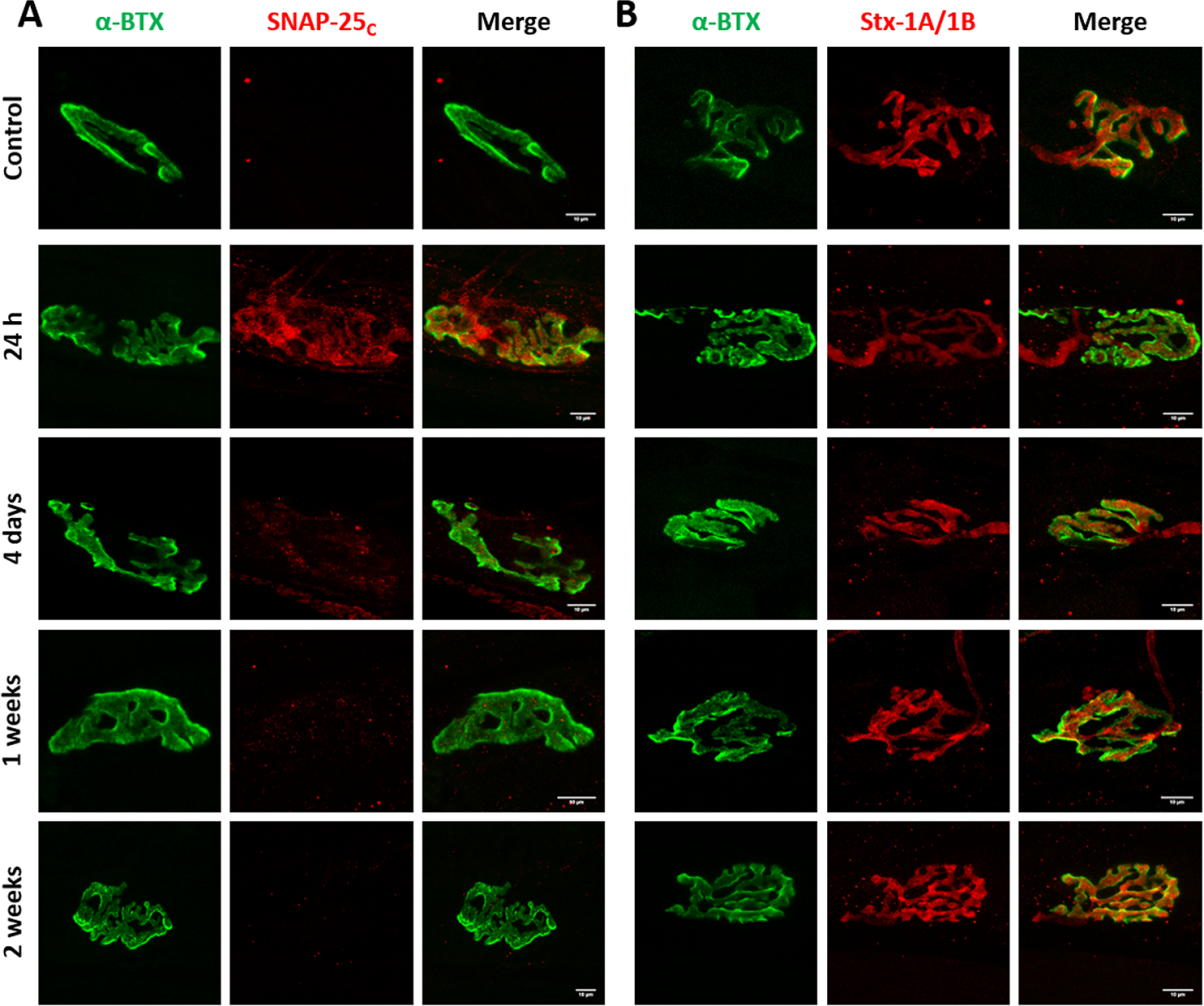
Imaging of cleaved SNAP-25 and syntaxin-1 in muscles treated with BoNT/C α-3W. Soleus muscles of mice treated with BoNT/C α-3W and used for the analysis of EJPs of Fig 5B were fixed immediately after the electrophysiological recordings and stained for **(A)** cleaved SNAP-25 (SNAP-25_c_) or **(B)** Syntaxin-1A/1B (Stx-1A/1B), both shown in red. NMJs were spotted with α-Bungarotoxin (α-BTX, in green). The first row of panels represents the staining of a control muscle. Scale bar, 10 μm.

**Fig 8:**
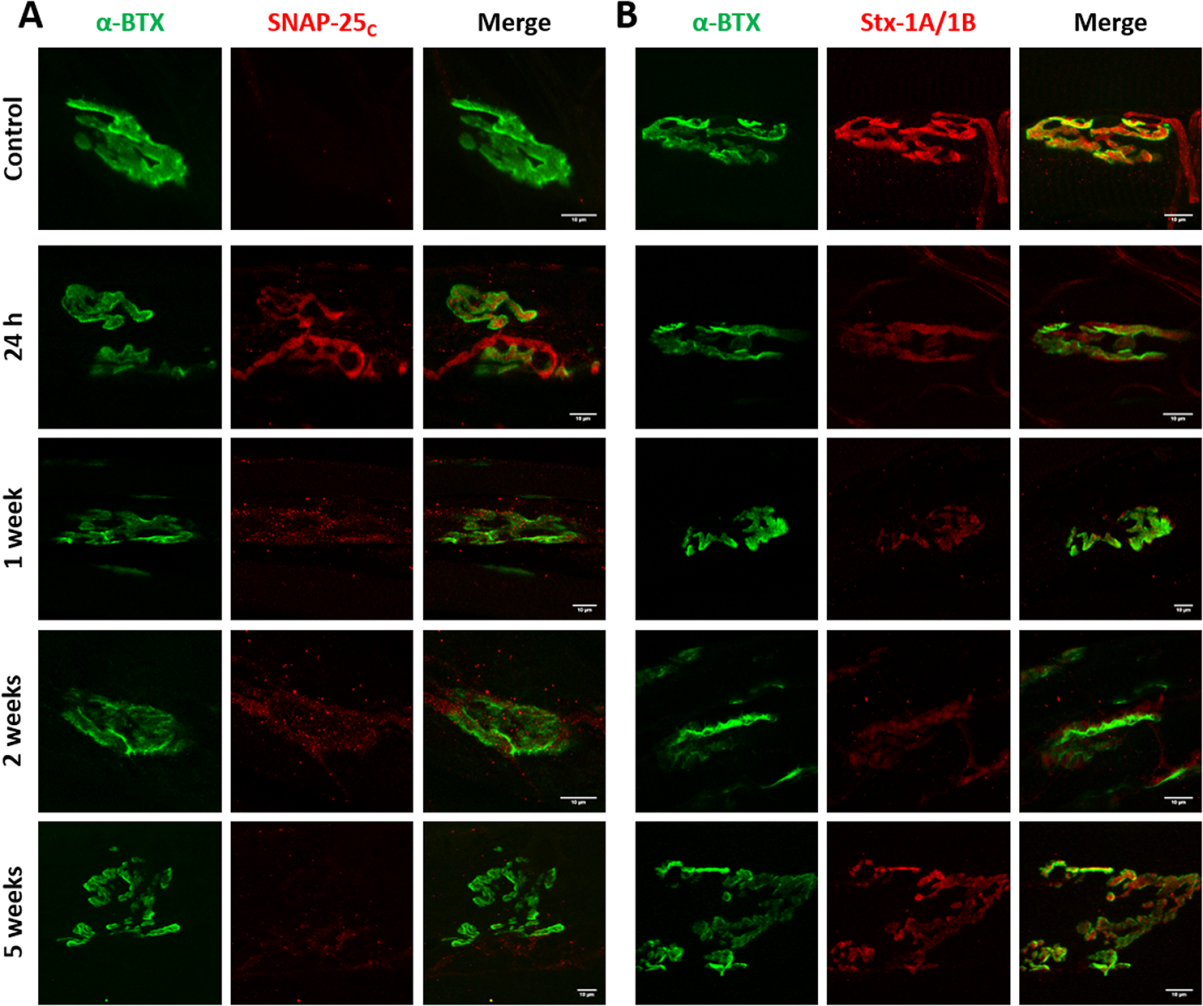
Imaging of cleaved SNAP-25 and syntaxin-1 in muscles treated with BoNT/C α-51. Soleus muscles of mice treated with BoNT/C α-51 and used for the analysis of EJPs of Fig 5B were fixed immediately after the electrophysiological recordings and stained for **(A)** cleaved SNAP-25 (SNAP-25_c_) or **(B)** Syntaxin-1A/1B (Stx-1A/1B), both shown in red. NMJ were spotted with α-Bungarotoxin (α-BTX, in green). The first row of panels represents the staining of a control muscle. Scale bar, 10 μm.

We hypothesized that lack of correlation between duration of paralysis and the relative potency of BoNT/C variants was due to different cleavage of SNAP-25 and syntaxin. To test this hypothesis, we stained soleus muscles of mice treated with wild type and mutant BoNT/C toxins for cleaved SNAP-25. BoNT/C-wt induced long-lasting SNAP-25 cleavage, which was reduced only at a later stage, when neurotransmission has significantly recovered (Fig 6A). Two weeks after toxin treatment, we also observed NMJ fragmentation, extensive nerve terminal sprouting, and synaptic remodeling, as previously reported [50]. Interestingly, cleaved SNAP-25 was detected all along sprout membranes and within the presynaptic side of newly formed nerve-muscle contacts. Following BoNT/C α-3W treatment, cleaved SNAP-25 was clearly detectable 24 hours after injection, when neurotransmission is completely blocked, but disappeared within four days, indicating that SNAP-25 1–198 has a half-life of <4 days (Fig 7A). NMJs displayed neither signs of postsynaptic remodeling nor nerve sprouting. These results suggest that SNAP-25 cleavage is associated with a nerve terminal blockage and that muscle paralysis persists as long as SNAP-25 is cleaved. On the other hand, Fig 8A shows that in muscles treated with BoNT/C α-51, cleaved SNAP-25 is evident only at 24 hours after injection and barely detectable at one and two weeks after. This indicates that SNAP-25 proteolysis occurs only at the very beginning of the time course and, consequently, the long lasting neurotransmission impairment induced by BoNT/C α-51 cannot be ascribed to SNAP-25 cleavage. Therefore, we stained injected muscles for syntaxin-1A/1B. Control NMJs displayed an intense and widespread staining characterized by large puncta of syntaxin-1B, the isoform prevalently expressed at the NMJ [51] (Figs 6B, 7B and 8B). BoNT/C and mutant toxins caused a large, though incomplete, loss of signal and the complete disappearance of syntaxin-1B clusters. This effect, which we interpreted as syntaxin-1B cleavage, was prolonged for BoNT/C-wt and it was not recovered even five weeks after injection (Fig 6B). In the case of BoNT/C α-3W, loss of syntaxin-1B was obvious only 24 hours after toxin injection with return to control levels between 7 to 14 days, indicating re-synthesis of syntaxin within 4 days and absence of LC/C α-3W activity (Fig 7B). BoNT/C α-51 caused a loss of syntaxin-1B similar to BoNT/C-wt and even 35 days post-injection there were no signs of recovery (Fig 8B). Importantly, nerve terminals underwent remodeling with a time course and an intensity comparable to the BoNT/C-wt-treated muscles, indicating a long-term impairment of NMJs. Considering that syntaxin re-synthesis is rapid (Fig 7B), these results suggest that the long lasting effect of BoNT/C α-51 is due to a persistent cleavage of syntaxin-1B and that LC/C α-3W activity has a very short half-life *in vivo*. Moreover, it is interesting to note that syntaxin proteolysis by LC/C α-51 also occurs in a phase in which SNAP-25 is not cleaved anymore (Fig 8B) which might be ascribed to the >10-fold higher EC_50_ for SNAP-25 (EC_50Stx_ 0.5 nM vs EC_50SNAP-25_ >5 nM).

Altogether, electrophysiological measurements and nerve terminal analyses indicate that the cleavage of SNAP-25 is necessary for a complete neuromuscular block and muscle paralysis, while the proteolysis of syntaxin-1B induces a significant, though incomplete, impairment of neurotransmission.

### A low dose of BoNT/C α-51 accounts for a prolonged modulation of neurotransmission at the neuromuscular junction

**Fig 9:**
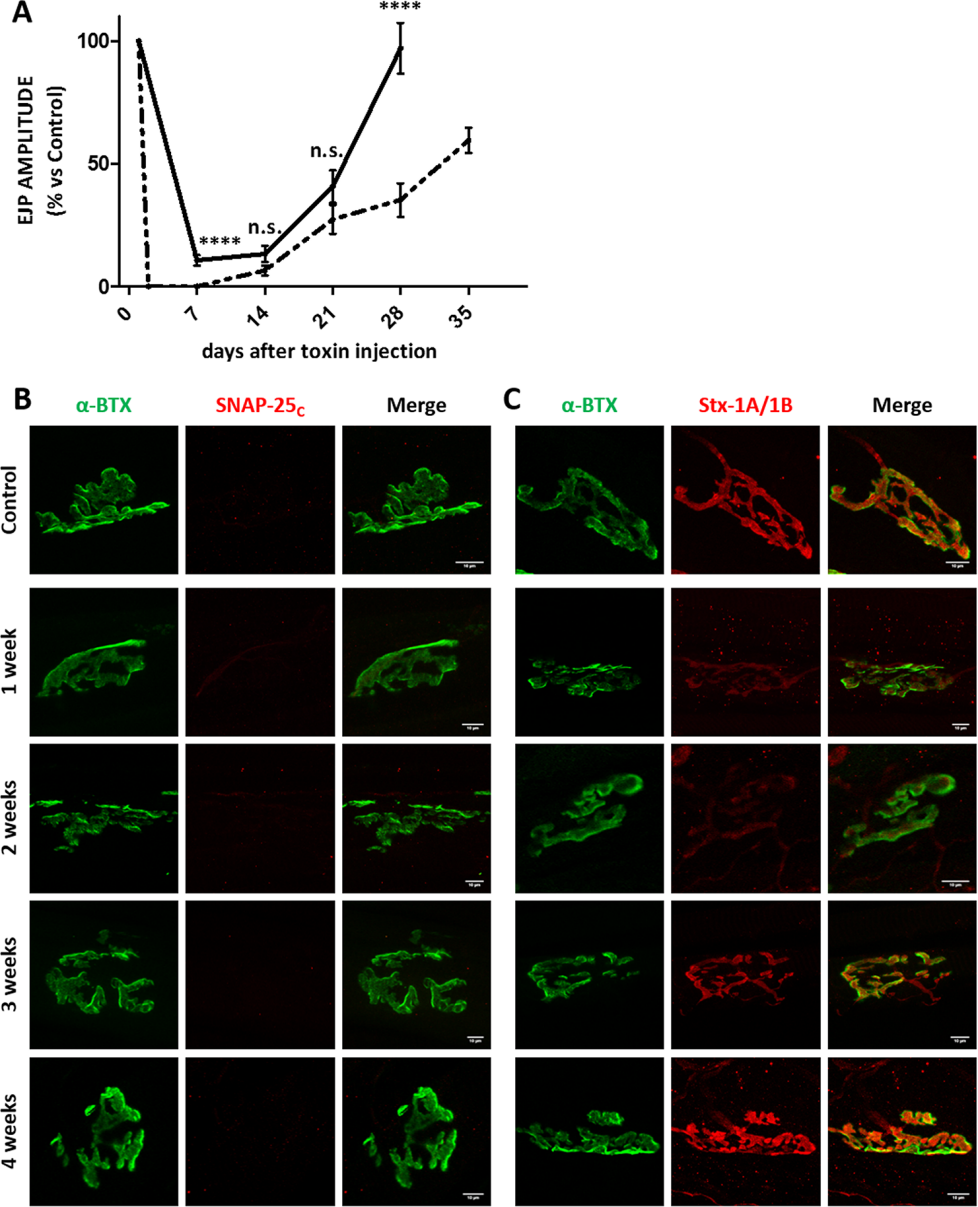
Time course of neurotransmission recovery in soleus muscles upon a local injection of a low dose of BoNT/C α-51. **(A)** The black trace shows the analysis of evoked post synaptic junction potentials (EJP) on soleus muscles injected intramuscularly with 10 ng/kg of BoNT/C α-51. At indicated time points soleus muscles were collected and processed for the electrophysiological recordings of EJPs, as previously reported [27]. Data are presented as a percentage of EJPs of control muscles. Each point represents an average EJP amplitude obtained from at least 45 muscle fibers from three different mice per condition. Error bars represent SEM. As a comparison, dotted trace shows the time course of EJP recovery obtained with 1 LD_50_ of BoNT/C α-51. Statistical significance at each time point was determined by a Student's t-test comparing the mean values (**** p<0.0001, n.s. not significant). Error bars represent SEM. **(B and C)** Soleus muscles coming from the EJP analyses were fixed and stained for (B) cleaved SNAP-25 (SNAP-25_c_) or (C) Syntaxin-1A/1B (Stx-1A/1B), both shown in red. NMJ were spotted with α-Bungarotoxin (α-BTX, in green). The first row of panels represents the staining of a control muscle.

Since the previous experiments showed that SNAP-25 cleavage occurs only with large amounts of mutant toxins, we reasoned that a low dose might induce exclusive proteolysis of syntaxin-1B. In this way, motor nerve activity should be attenuated without complete abrogation of neurotransmission. To test this hypothesis, we opted to use BoNT/C α-51 because of its low activity against SNAP-25 and its long half-life. Toxin was injected in the hind-limb at a dose of 10 ng/kg, which corresponds to about 1/75 of its LD_50_ and 13-fold LD_50_ of BoNT/C-wt. Interestingly, this “low dose” of BoNT/C α-51 did not result in a visible neuroparalysis (i.e., DAS score 0). Rather BoNT/C α-51 caused a substantial decrease of EJP amplitude in injected muscles, which lasted for almost one month before returning to control levels (Fig 9A). Importantly, no cleavage of SNAP-25 was detected during the entire time course (Fig 9B), while a significant loss of syntaxin-1B staining occurred, especially within the first two weeks after injection (Fig 9C). Thereafter, syntaxin-1B expression recovered together with neurotransmission, suggesting that nerve terminal activity (EJP amplitude) is proportional to the amount of syntaxin-1B. In addition, we found a reduced atrophy of muscles injected with the low dose of BoNT/C α-51 relative to muscles injected with either 1 LD_50_ of BoNT/C α-51 or 1 LD_50_ of BoNT/C-wt. These results indicate that BoNT/C α-51 can persistently modulate nerve terminal activity without compromising the overall activity of muscles.

## Discussion

Among the many botulinum neurotoxins characterized so far, BoNT/C is unique in that it cleaves two neuronal SNARE proteins, i.e. SNAP-25 and syntaxins. Although this parallel activity was demonstrated over 20 years ago in cultured neurons [24], it has never been reported at the NMJs nor was it clear which proteolytic event by BoNT/C causes neuroparalysis. Our results show that BoNT/C cleaves both substrates at the NMJ, and that the key determinant of potency and lethality is the proteolysis of SNAP-25 rather than syntaxin. We also report that the cleavage of syntaxin-1B (syntaxin-1A is not expressed at the NMJ [51]) does not cause complete block of the NMJ, although it accounts for a substantial impairment of neurotransmission efficiency. Such a result is surprising considering the current view of the SNARE-mediated mechanism of neuroexocytosis [52], but it is supported by previous reports showing that: i) BoNT/C paralysis at NMJ can be reversed by 3,4-diaminopyridine [50], consistent with a neuroparalytic effect lying on SNAP-25 cleavage rather than syntaxin, like for BoNT/A1 [53]; ii) Syntaxin-1B knock out mice have a very limited life span, yet they survive for a couple of weeks after birth, implying that neuromuscular transmission is viable [54, 55]; iii) Syntaxin-1B deficiency reduces but does not abolish NMJ capacity of neurotransmitter release [55]. A likely explanation is the compensation of syntaxin-1B knock out/proteolysis at the NMJ by other syntaxin isoforms. In knock out mice a minor expression of syntaxin-1A was reported, which might occur as a compensatory mechanism [55]. In the present study, this possibility has to be discarded as syntaxin-1A would also be substrate of the toxins. On the other hand, many non-cleavable syntaxins exist [5], raising the possibility that a cognate isoform compensates for syntaxin-1 biochemical knock down, leading to a largely inefficient, yet functional neurotransmitter release.

As a general conclusion, syntaxin-1B proteolysis does not seem to be critical for the acute neuroparalytic action of BoNT/C, which instead relies on SNAP-25 cleavage. Nonetheless, syntaxin cleavage may contribute to delaying the recovery process. In fact, fusion of synaptic vesicles with the plasma membrane depends on the incorporation of multiple SNARE complexes into SNARE super-complexes [56]. Since cleavage by BoNT/C occurs at the very C-terminus and frees syntaxin from its transmembrane domain [5, 13, 14], it may be speculated that the SNARE motif of cleaved-syntaxins are incorporated within SNARE super-complexes, and negatively modulate vesicle fusion. This effect may be added to the long known effect of BoNT/C (and BoNT/A) cleaved-SNAP-25 in modulating neuroexocytosis [5, 56].

We were surprised to find that BoNT/C mutants exert different neurodegenerative effects in cultured neurons. This effect was previously attributed to cleavage of SNAP-25 and syntaxin-1A/1B, and to the complete elimination of one of them [40, 41]. However, we found that *in vitro* neurodegeneration triggered by BoNT/C-wt and BoNT/C α-51 occurs even if a small portion of syntaxin-1A/1B is resistant to cleavage. Moreover, BoNT/C α-3W is not cytotoxic even if it displays proteolytic activity against SNAP-25 and syntaxin-1A/1B to an extent equal, if not superior, to BoNT/C α-51. These results indicate that SNARE cleavage may be not directly implicated in BoNT/C-mediated neurodegeneration, at least in CGNs. An intriguing alternative explanation may be the proteolysis of an additional substrate, still recognized by BoNT/C-wt and BoNT/C α-51, but not by BoNT/C α-3W.

BoNT/C neurodegeneration does not occur at the NMJ *in vivo* in mice [50] and humans [48, 57, 58]. A plausible explanation as to why neurodegeneration occurs only in cell culture may be found in the different mode of entry of BoNT/C at the NMJ and in cultured neurons. *In vivo*, like the other BoNTs, BoNT/C entry is restricted to unmyelinated areas of the nerve terminals as axons are covered and protected by a tight nerve-blood-barrier. Neurons in culture are instead not myelinated and fully exposed to the action of all toxins residing in the culture medium. Accordingly, BoNT/C may affect neuronal compartments that are not accessible *in vivo*. In any case, we show here that poisoned nerve terminals do not degenerate at any time after toxin injection, a relevant finding considering that very high amounts of BoNT/C α-51 had been locally injected (1 LD_50_ ≥ 15 ng/mouse). Rather, poisoned nerve terminals activate and set in motion a profound remodeling of the NMJ as an attempt to bypass the functional block of the synapse, similarly to what is observed upon BoNT/A1 and BoNT/B1 intoxication [25, 27, 50, 59]. Therefore, our results suggest that the use of BoNT/C in humans would be safe, and that BoNT/C α-51 may be a suitable candidate for this purpose. In fact, used at a dose comparable to BoNT/C-wt, this toxin provides a persistent modulation of nerve terminal activity without causing the complete paralysis of the muscle, a relevant finding for therapeutic and cosmetic applications. BoNT/C α-51 would likely be ideal for applications characterized by a narrow therapeutic window, when an optimal modulation of nerve terminal hyperactivity is usually difficult to achieve without causing significant muscle weakening, as in focal dystonia [5, 60]. Moreover, considering the low systemic toxicity in mice, this toxin may be suitable in clinical conditions requiring considerable amounts of BoNT, like the treatment of large muscles in post-stroke spasticity [5, 61, 62]. As for other BoNTs [29, 57, 58, 63], electrophysiological testing on human volunteers can be used to assess the time course of action and the susceptibility of human muscles to BoNT/C α-51 (or syntaxin-specific BoNTs). These preliminary analyses would reveal a dose-response window and may be essential in evaluating the therapeutic potential of the toxin as well as its safety margin and immunogenicity [5].

In conclusion, this work highlights that minimal changes can functionally impact BoNT biological activity, and suggests that inspection of structure-activity relationships may be used to generate tailor-made toxins with *ad hoc* pharmacological properties to improve the present applications and expand the clinical landscape of BoNT pharmacotherapy [41, 46, 64, 65].

## Materials and methods

### Reagents

Native BoNT/C and BoNT/A1 were purified as previously described [66, 67]. Cytosine β-D-arabinofuranoside hydrochloride (C6645), DNAse I from bovine pancreas (DN25), poly-L-lysine hydrobromide (P1274) and trypsin (T4799) were from Sigma Aldrich. μ-Conotoxin GIIIB is from Alomone, Jerusalem, Israel. Primary antibodies: anti-SNAP-25 (SMI81, ab24737) was from Abcam. Anti-SNAP-25 (cleaved) and syntaxin-1A/1B polyclonal antibodies were produced in our laboratory and previously characterized [37, 68]. Secondary antibodies conjugated to HRP were from Calbiochem; secondary antibodies for immunofluorescence conjugated to Alexa Fluorophores 488 or 555 and α-Bungarotoxin conjugated to Alexa 647 were from Thermo Scientific, Waltham, MA, USA.

### Production of recombinant BoNT/C and SNARE substrates

Full-length BoNT/C (GenBank: X53751.1) and BoNT/C LC (aa 1–430) as well as the mutants thereof were produced, the former under biosafety level 2 containment (project number GAA A/Z 40654/3/57), in *E*. *coli* strain M15pREP4 (Qiagen, Hilden, Germany) during 15 h of induction at 21°C and purified on StrepTactin-sepharose matrix (IBA GmbH, Göttingen, Germany) and Ni^2+^-nitrilotriacetic acid-agarose matrix (Qiagen), respectively, according to the manufacturers’ instructions. Aliquots of BoNT/C derivatives (in 100 mM Tris, pH 8.0) and of BoNT/C LC derivatives (dialyzed against toxin assay buffer (150 mM potassium glutamate, 10 mM HEPES-KOH, pH 7.2), were frozen in liquid nitrogen, and kept at -70°C.

Recombinant substrate proteins, rat SNAP-25His6 [69] and a syntaxin fusion protein comprising an N-terminal His6-tag followed by the Halo-tag, rat syntaxin 1A aa 183–259, luciferase, and a C-terminal Strep-tag, were produced using the *E*. *coli* strains M15 pREP4 and BL21-DE3 (Stratagene Europe, Ebsdorfergrund, Germany), respectively, purified via His6- or His6- and Strep-tag, dialyzed against toxin assay buffer or PBS, pH 7.4, supplemented with 7% (w/v) sucrose, and finally frozen in liquid nitrogen.

Radiolabeled substrates were generated by *in vitro* transcription/translation using the plasmids pSNAP-25his6 and pET29-HASyn(183–259)LS, the SP6/T7 coupled TNT reticulocyte lysate system (Promega), and [^35^S]methionine (370 KBq/μL, >37 TBq/mmol, Hartmann Analytic, Braunschweig, Germany) according to the manufacturer´s instructions.

Concentrations of *E*. *coli* expressed proteins were determined subsequent to SDS-PAGE and Coomassie blue staining by using a LAS-3000 imaging system (Fuji Photo Film), the AIDA 3.51 program, and various known concentrations of BSA. The extent of hydrolytic activation of full-length BoNT/C by *E*. *coli* proteases was 81% (wild type), 73% (α-51), and 79% (α-3W).

### *In vitro* proteolytic activity

Cleavage assays were conducted using 10 μM SNAP-25 or 1 μM syntaxin fusion protein, respectively, each 1 μL of transcription/translation mixture of the respective substrate as [^35^S]-methionine-labeled protein, and purified LC/C derivative at 1 to 3 μM final concentrations in 10 μL. Incubation was done for 60 min at 37°C in toxin assay buffer. Reactions were stopped by the addition of an equal volume of double-concentrated sample buffer (120 mM Tris-HCl, pH 6.75, 10% (v/v) β-mercaptoethanol, 4% (w/v) SDS, 20% (w/v) glycerol, 0.014% (w/v) bromphenol blue) and then subjected to SDS-PAGE using 10% or 15% tris/glycine gels (the latter using acrylamide/bis-acrylamide in 73.5:1 ratio). Subsequently, gels were dried and radiolabeled proteins were visualized employing a FLA-9000 phosphorimager (Fuji Photo Film, Co., Ltd., Tokyo, Japan). Quantification of cleavage was done by means of the radiolabeled substrates by phosphorimaging using the Multigauge 3.2 software (Fuji Photo Film).

### Neuronal cultures and intoxication assay

Primary cultures of rat cerebellar granule neurons (CGNs) were prepared from 6- to 8-day-old rats as previously described [26]. Briefly, cerebella were isolated, mechanically disrupted and trypsinized in the presence of DNase I. Cells were then collected and plated into 24 well plates pre-coated with poly-L-lysine (50 μg/ml) at a cell density of 4x10^5^ cells per well. Cultures were maintained at 37°C, 5% CO_2_, 95% humidity in BME (Basal Medium Eagle) supplemented with 10% fetal bovine serum, 25 mM KCl, 2 mM glutamine and 50 μg/ml gentamicin (hereafter indicated as complete culture medium). To arrest growth of non-neuronal cells, cytosine arabinoside (10 μM) was added to the complete culture medium 18–24 h after plating.

CGNs at 6–8 days *in vitro* (DIV) were incubated with increasing concentrations (from 0.01 nM to 5 nM) of the indicated BoNT/C in complete culture medium for 12 or 24 hours at 37°C. The specific proteolytic activity against SNAP-25 and syntaxin-1A/1B was evaluated via immunoblotting with antibodies that recognize both the intact and the truncated form of the two proteins.

### Immunoblotting

Cells were directly lysed with Laemmli sample buffer containing protease inhibitors (Roche). Cell lysates were loaded onto NuPage 12% Bis-Tris gels (Life technologies) and separated by electrophoresis in MOPS buffer (Life technologies). Proteins were transferred onto Protran nitrocellulose membranes (Whatman) and saturated for 1 h in PBS-T (PBS, 0.1% Tween 20) supplemented with 5% non-fatty milk. Incubation with primary antibodies was performed overnight at 4°C. The membranes were then washed three times with PBS-T and incubated with appropriate secondary antibodies for 1 h. Membranes were washed three times with PBS and proteins revealed either with an Odyssey imaging system (LI-COR Bioscience) or with an Uvitec gel doc system (Uvitec Cambridge).

### Immunocytochemistry

CGNs were seeded onto 13 mm round glasses in 24-well plates at a cell density of 4x10^5^ cells per well. CGNs at 6–8 DIV were incubated for the indicated time and concentration of toxin in complete culture medium at 37°C. After treatment, neurons were fixed for 10 min with 4% (w/v) paraformaldehyde in PBS and stained with an antibody against cleaved SNAP-25 and an antibody against neurofilament-200 (NF200). Coverslips were mounted using Fluorescent Mounting Medium (Dako) and examined with a Leica SP5 confocal microscope (Leica Microsystems, Wetzlar, Germany) equipped with 100X HCX PL APO NA 1.4 objective.

### Mouse phrenic nerve (MPN) hemidiaphragm assay

The MPN assay was performed as described previously [43, 45]. To limit the consumption of mice, the left and right phrenic nerve hemidiaphragms were excised from female mice of strain RjHan:NMRI (18–25 g, Janvier, St Berthevin Cedex, France) and placed in an organ bath containing 4 ml of Earle's Balanced Salt Solution. The pH was adjusted to 7.4, and oxygen saturation was achieved by gassing with 95% O_2_ and 5% CO_2_. The phrenic nerve was continuously electro-stimulated at a frequency of 1 Hz with a pulse duration of 0.1 ms and a current of 25 mA to achieve maximal contraction amplitudes. Isometric contractions were recorded with a force transducer (Scaime, Annemasse, France) and the software VitroDat (Föhr Medical Instruments GmbH (FMI), Seeheim, Germany). The resting tension of the hemidiaphragm was approximately 10 mN. In each experiment, the preparation was first allowed to equilibrate for 15 min under control conditions. Then, the buffer was exchanged to 4 ml of Earle's Balanced Salt Solution supplemented with 0.1% BSA and varying BoNT/C dilutions. The previously reported calibration curve determined for recombinant, *E*. *coli* host activated BoNT/C (y (BoNT/C; 15, 50, 70, 100 and 233 pM) = 148.95x^-0.2089^; R^2^ = 0.9806) was used to calculate the residual potency of BoNT/C mutants. The resulting paralytic half-times of BoNT/C mutants were converted to the corresponding concentrations of wild type BoNT/C, using the equation mentioned above. The toxicities were finally expressed relative to wild type BoNT/C.

### Lethality assay

Swiss-Webster adult female CD1 mice (20–24 grams) were housed under controlled light/dark conditions, and food and water were provided *ad libitum*. All experiments were performed in accordance with the European Community Council Directive n° 2010/63/UE and approved by the Italian Ministry of Health. LD_50_ were determined by injecting different doses of BoNT/C-wt or BoNT/C α-3W or BoNT/C α-51 diluted in 0.9% NaCl 0.2% gelatin. Toxins were prepared at a given concentration and mice were injected intraperitoneally with different volumes according to their body weight in order to reach the indicated doses. Mice were monitored every 4 hours for 96 hours, when the experiment was considered to be concluded.

### Digit abduction score (DAS) assay

Swiss-Webster adult female CD1 mice weighing 20–24 g were injected in the left hind limb with 1xLD_50_ of BoNT/C-wt (0.75ng/kg) or BoNT/C α-3W (150 ng/kg) or BoNT/C α-51 (750 ng/kg) diluted in 0.9% NaCl with 0.2% gelatin. Neuroparalysis was assessed daily according to the Digit Abduction Score (DAS) assay scale, as previously reported [49].

### Electrophysiological recordings of evoked junction potential

Mice were injected in the left hind limb as described for the DAS assay with indicated doses. At scheduled times, mice were sacrificed by anesthetic overdose coupled to cervical dislocation and the soleus muscle dissected. Electrophysiological recordings were performed in oxygenated Krebs-Ringer solution, using intracellular glass microelectrodes (WPI) filled with 1 M KCl and 2 M CH_3_COOK. Evoked junction potentials (EJP) were recorded in current-clamp mode, starting from resting membrane potential of -70 mV, adjusted with direct current injection if needed. EJPs were elicited by supramaximal nerve stimulation at 0.5 Hz, using a suction microelectrode connected to a S88 stimulator (Grass, Warwick, RI, USA). Muscle contraction was prevented by 1 μM μ-Conotoxin GIIIB (Alomone, Jerusalem, Israel). Signals were amplified with intracellular bridge mode amplifier (BA-01X; NPI, Tamm, Germany), sampled using a digital interface (NI PCI-6221; National Instruments, Austin, TX, USA) and recorded by means of electrophysiological software (WinEDR; Strathclyde University, Glasgow, Scotland, UK). EJPs measurements were carried out with Clampfit software (Molecular Devices, Sunnyvale, CA, USA). EJPs represent the average value obtained analyzing at least three muscles (15 fibers/muscle) for each condition at each time-point and reported as a percentage with respect to control muscles.

### Imaging of neuromuscular junctions

Immediately after electrophysiological recording, soleus muscles were fixed in 4% paraformaldehyde in PBS for 10 min at RT. Each muscle was then separated in bundles of about 20–40 fibers to facilitate the following steps, in particular antibody penetration. In the case of explanted and intoxicated hemidiaphragms, upon completion of paralysis, muscles were fixed for 30 minutes. Samples were quenched in 50 mM NH_4_Cl in PBS and treated for 2 h with a blocking solution (15% v/v goat serum, 2% w/v BSA, 0.25% w/v gelatin, 0.2% w/v glycine in PBS, 0.5% Triton X-100) to saturate and permeabilize nerve terminals. Thereafter, incubation with the following primary antibodies was carried out for at least 48 h in blocking solution with either anti-cleaved SNAP-25 or anti-Syntaxin-1A/1B. Muscles were then extensively washed and incubated with a secondary antibody conjugated with Alexa-555 diluted in blocking solution supplemented with α-Bungarotoxin conjugated to Alexa 647 to counterstain post-synaptic nicotinic acetylcholine receptors. Images were collected with a Leica SP5 confocal microscope (Leica Microsystems, Wetzlar, Germany) equipped with 100X HCX PL APO NA 1.4 objective. Laser excitation line, power intensity, and emission range were chosen according to each fluorophore in different samples to minimize bleed-through.

### Ethics statements

All experiments were performed in accordance with the Italian laws and policies (D.L. n°26 14^th^ March 2014) and with the guidelines established by the European Community Council Directive n° 2010/63/UE and approved by the veterinary services of the University of Padova (O.P.B.A.—Organismo Preposto al Benessere degli Animali) (protocol 359/2015).

## Supporting information

S1 Fig Mutations conferring to BoNT/C specificity for syntaxins. Space-filling representation of BoNT/C LC (PDB entry 2QN0) with highlighted triple mutations for syntaxin selectivity [32]: S51T/R52N/N53P (BoNT/C α-51) in green and L200W/M221W/I226W (BoNT/C 0078-3W) in red. Blue spot shows the metalloprotease active site.
(TIF)

S2 Fig SDS-PAGE analysis of the different BoNT/C toxins used in the study. From left to right, 250 nanograms of either native BoNT/C-wt, or recombinant BoNT/C-wt, BoNT/C α-51 or BoNT/C α-3W were loaded in a 12% gel under reducing conditions and revealed by Coomassie staining. The extent of hydrolytic activation of full-length BoNT/C by *E*. *coli* proteases was 81% (wild type), 73% (α-51), and 79% (α-3W). The lower purity of α-51 does not compromise its biological activity as deduced by the very similar EC_50Stx_ of this toxin with respect to BoNT/C α-3W and BoNT/C-wt in cultured neurons.
(TIF)

S3 Fig The cleavage of SNAP-25 by BoNT/C mutants in CGNs does not increase by prolonging the incubation time to 24 hours. CGNs were treated as in Fig 1 but incubation was prolonged to 24 hours. The cleavage of syntaxin-1A/1B and SNAP-25 was assayed by western blot using two antibodies recognizing both the intact and the cleaved forms of the proteins.
(TIF)

S4 Fig SNAP-25 cleaved by BoNT/C is recognized by an antibody raised against SNAP-25 cleaved by BoNT/A1. CGNs were treated with BoNT/A1 (0.1 nM) or BoNT/C-wt (0.1 nM) in normal culture medium at 37°C for 3 hours. Thereafter cells were fixed and stained with an antibody raised against SNAP-25 segment 185–197 (red) [37], corresponding to the C-terminus generated by BoNT/A1 cleavage (SNAP-25_c_). The antibody against neurofilament-200 (NF200, in green) is used as control staining. Scale bar, 10 μm.
(TIF)

S5 Fig BoNT/C mutants display a different cytotoxic effect on cultured neurons. CGNs were treated as in Fig 2 but incubation was prolonged to 24 hours. Neurons were then fixed and stained with an antibody against cleaved SNAP-25 (SNAP-25_c_, in red) and neurofilament-200 (NF200, in green). Cytotoxicity was evaluated following the appearance of varicosities along neurites and the loss of NF200 staining. Images are representative of at least three independent experiments. Scale bar, 10 μm.
(TIF)
