# Supplementary figures and images for: Botulinum neurotoxin C mutants reveal different effects of syntaxin or SNAP-25 proteolysis on neuromuscular transmission
Source: PLoS Pathog. 2017 Aug 11;13(8):e1006567. doi: 10.1371/journal.ppat.1006567 (PMC5568444; doi:10.1371/journal.ppat.1006567)

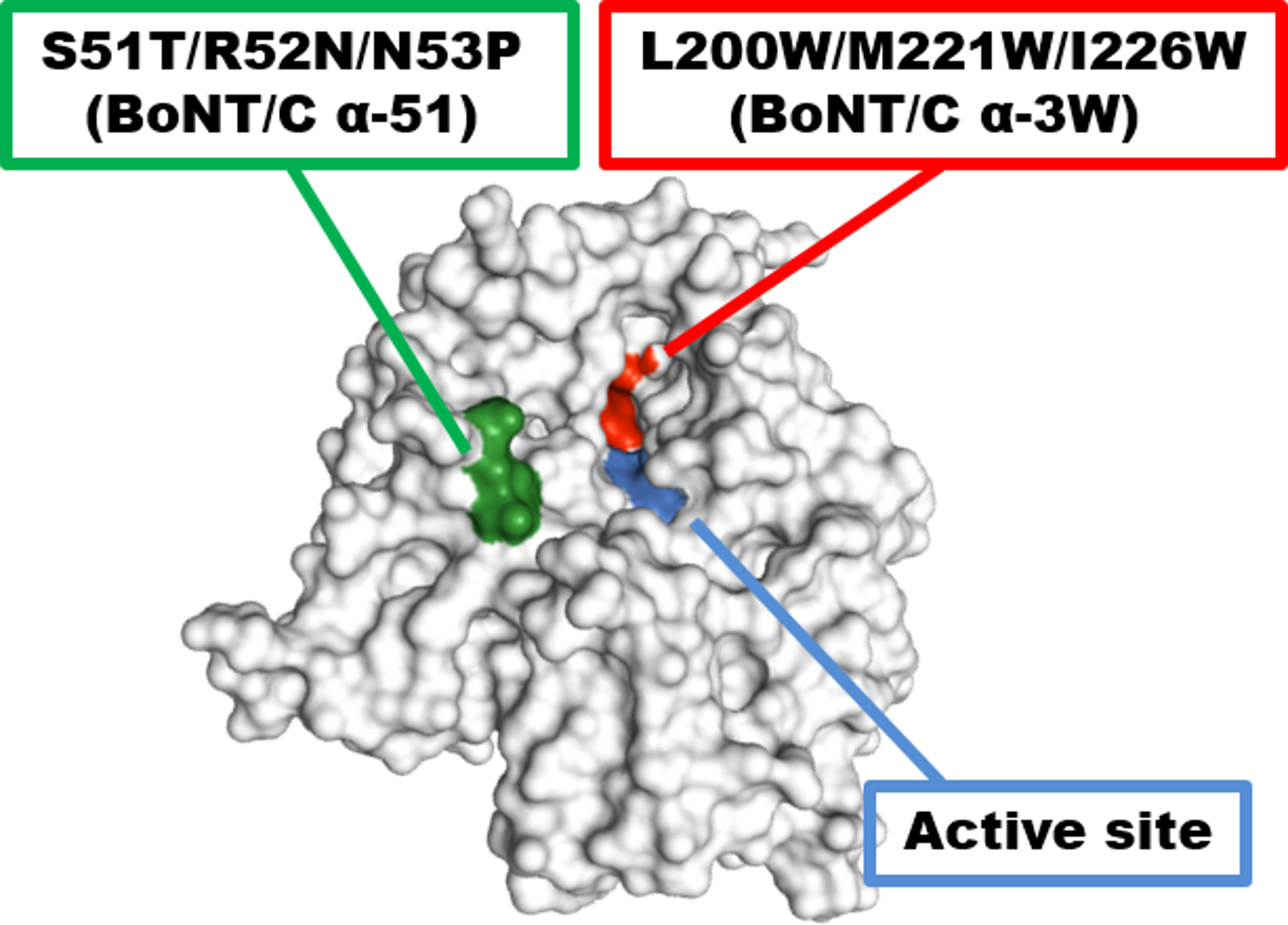

Supplement: S1 Fig — Space-filling representation of BoNT/C LC (PDB entry 2QN0) with highlighted triple mutations for syntaxin selectivity [32]: S51T/R52N/N53P (BoNT/C α-51) in green and L200W/M221W/I226W (BoNT/C 0078-3W) in red. Blue spot shows the metalloprotease active site. (TIF) [file ppat.1006567.s001.tif]

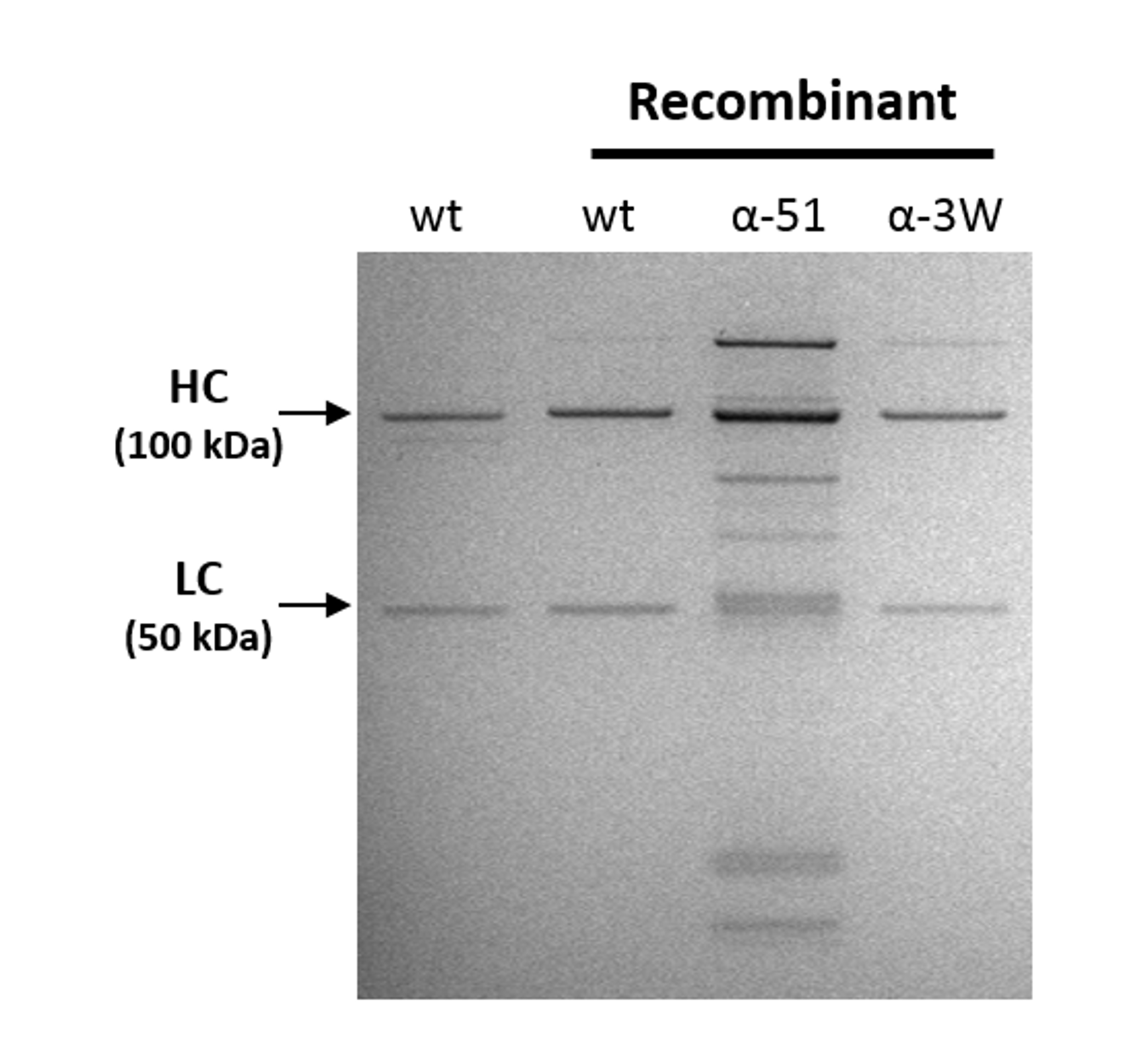

Supplement: S2 Fig — From left to right, 250 nanograms of either native BoNT/C-wt, or recombinant BoNT/C-wt, BoNT/C α-51 or BoNT/C α-3W were loaded in a 12% gel under reducing conditions and revealed by Coomassie staining. The extent of hydrolytic activation of full-length BoNT/C by E. coli proteases was 81% (wild type), 73% (α-51), and 79% (α-3W). The lower purity of α-51 does not compromise its biological activity as deduced by the very similar EC50Stx of this toxin with respect to BoNT/C α-3W and BoNT/C-wt in cultured neurons. (TIF) [file ppat.1006567.s002.tif]

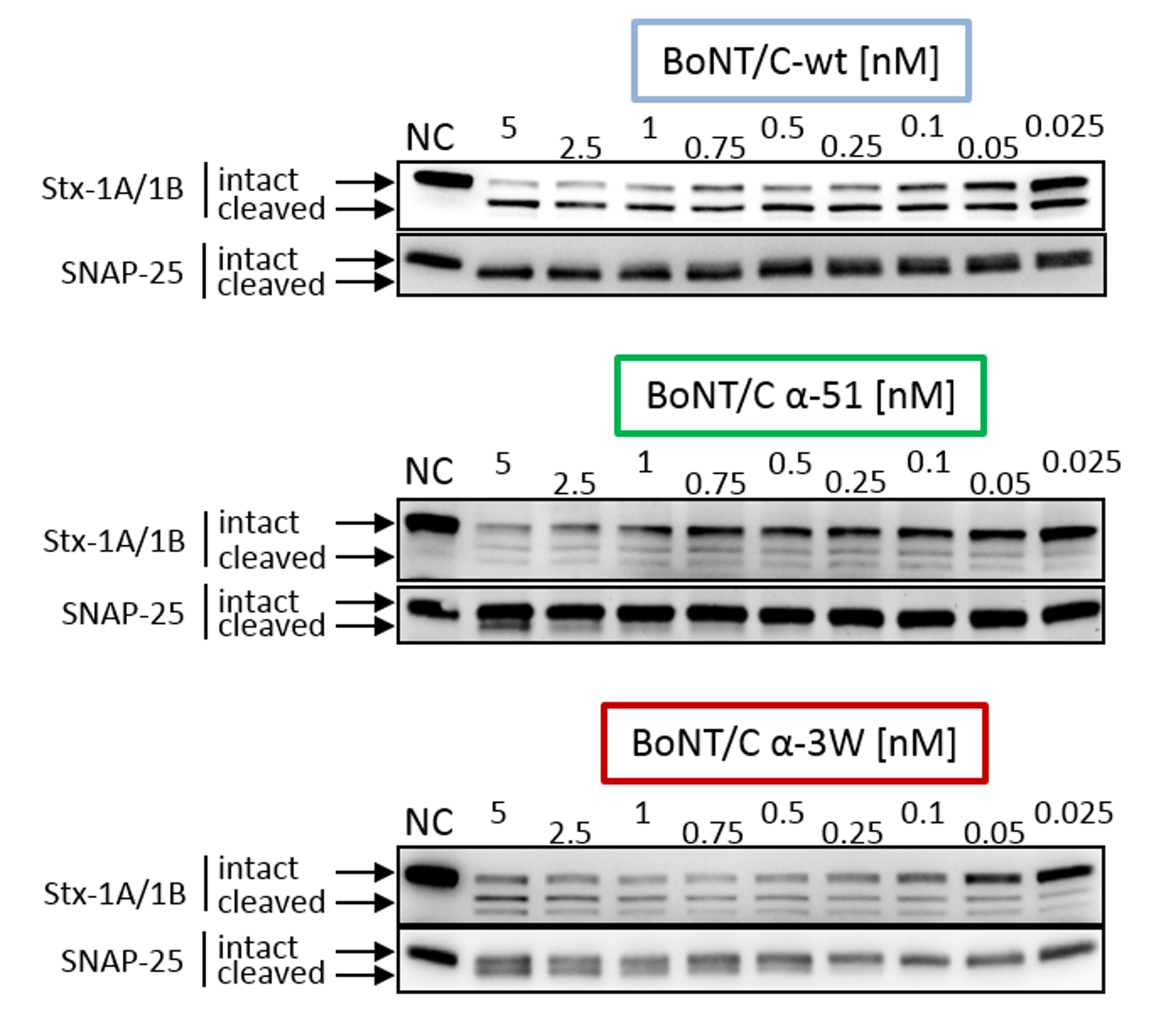

Supplement: S3 Fig — CGNs were treated as in Fig 1 but incubation was prolonged to 24 hours. The cleavage of syntaxin-1A/1B and SNAP-25 was assayed by western blot using two antibodies recognizing both the intact and the cleaved forms of the proteins. (TIF) [file ppat.1006567.s003.tif]

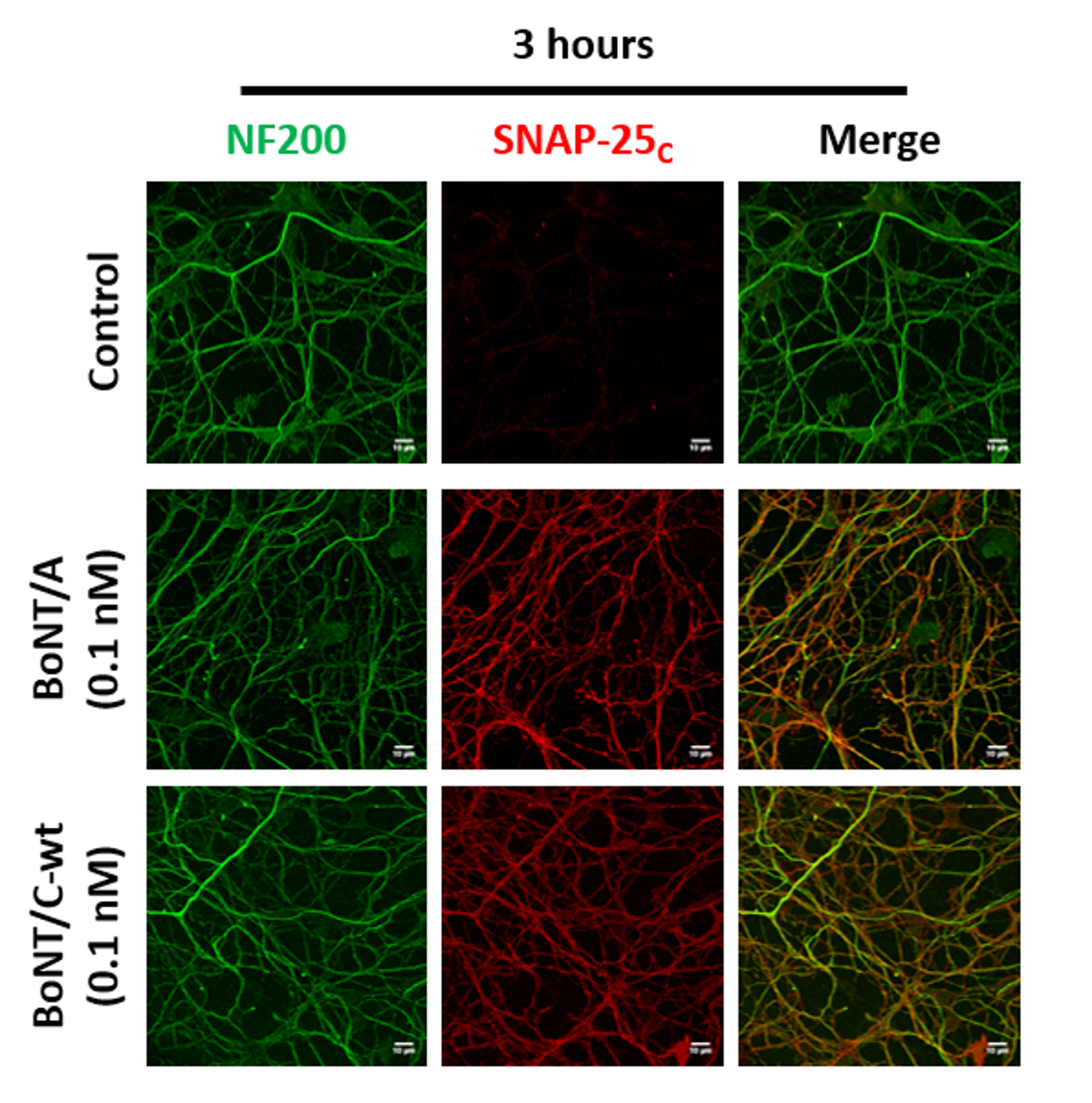

Supplement: S4 Fig — CGNs were treated with BoNT/A1 (0.1 nM) or BoNT/C-wt (0.1 nM) in normal culture medium at 37°C for 3 hours. Thereafter cells were fixed and stained with an antibody raised against SNAP-25 segment 185–197 (red) [37], corresponding to the C-terminus generated by BoNT/A1 cleavage (SNAP-25c). The antibody against neurofilament-200 (NF200, in green) is used as control staining. Scale bar, 10 μm. (TIF) [file ppat.1006567.s004.tif]

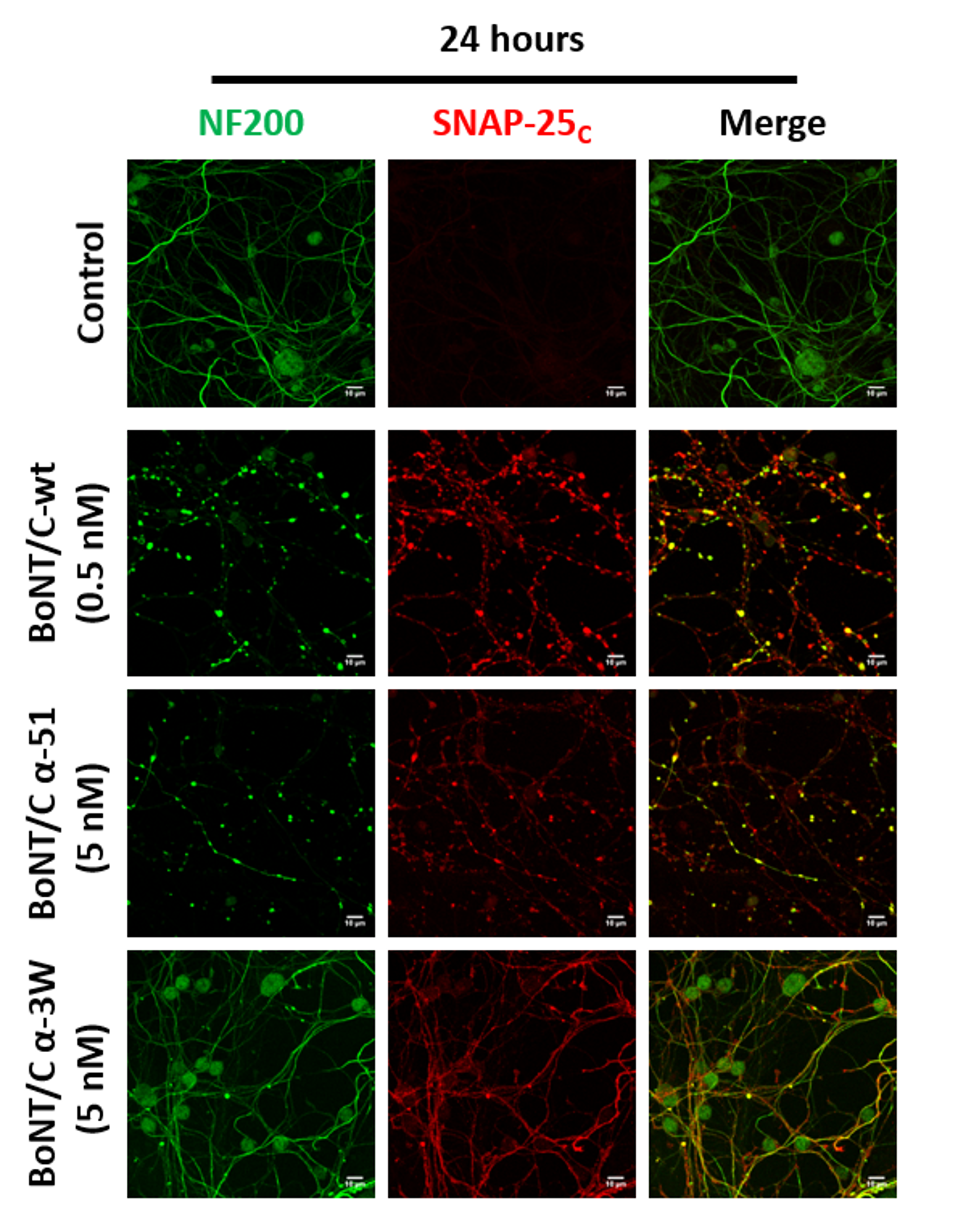

Supplement: S5 Fig — CGNs were treated as in Fig 2 but incubation was prolonged to 24 hours. Neurons were then fixed and stained with an antibody against cleaved SNAP-25 (SNAP-25c, in red) and neurofilament-200 (NF200, in green). Cytotoxicity was evaluated following the appearance of varicosities along neurites and the loss of NF200 staining. Images are representative of at least three independent experiments. Scale bar, 10 μm. (TIF) [file ppat.1006567.s005.tif]
